# Characterization of a Patch Antenna Sensor’s Resonant Frequency Response in Identifying the Notch-Shaped Cracks on Metal Structure

**DOI:** 10.3390/s19010110

**Published:** 2018-12-30

**Authors:** Liang Ke, Zhiping Liu, Hanjin Yu

**Affiliations:** School of Logistics Engineering, Wuhan University of Technology, 1178 Heping Ave., Wuhan 430063, China; keliang@whut.edu.cn (L.K.); yuhanjinwh@whut.edu.cn (H.Y.)

**Keywords:** patch antenna, sensor, structural health monitoring, crack identification, resonant frequency

## Abstract

Patch antenna sensor is a novel sensor that has great potential in structural health monitoring. The two resonant frequencies of a patch antenna sensor are affected by the crack on its ground plane, which enables it to sense the crack information. This paper presents a detailed characterization of the relationship between the resonant frequencies of a patch antenna sensor and notch-shaped cracks of different parameters, including the length, the orientation, and the center location. After discussing the principle of crack detection using a patch antenna sensor, a parametric study was performed to understand the response of the sensor’s resonant frequencies to various crack configurations. The results show that the crack parameters affect the resonant frequencies in a way that can be represented by the crack’s cutting effect on the sensor’s current flow. Therefore, we introduced a coefficient φ to comprehensively describe this interaction between the crack and the current distribution of the antenna radiation modes. Based on the definition of coefficient φ, an algorithm was proposed for predicting the resonant frequency shifts caused by a random notch-shaped crack and was verified by the experimental measurements. The presented study aims to provide the foundation for the future use of the patch antenna sensor in tracking the propagation of cracks of arbitrary orientation and location in metal structures.

## 1. Introduction

Metal structures are widely used in the fields such as mechanical equipment, civil infrastructure, aerospace facilities, and offshore platforms. In the long-term load bearing process, a variety of damage can be seen on metal structures. When the damage accumulates to a certain level, the structure’s load-carrying and anti-fatigue capacity will be impaired, which may lead to extremely serious consequences. To ensure the safe operation and prolong the lifespan of metal structures, a common method is to use sensors to monitor the structure’s health status. Since fatigue-related cracking is the major form of structural damage [1], a number of techniques have been developed for the purpose of crack identification. Vibration analysis [2,3] can be used for crack detection because the presence of crack would change the structure’s dynamic properties such as natural frequency and mode shape, which can give clues about the crack location and magnitude. However, the vibration characteristics are usually not sensitive to small-size local cracks. Strain-based analysis is another method to detect crack, and this is based on the fact that the emergence or extension of cracks would dramatically disturb the strain distribution in its vicinity. A very common tool to achieve this is an optical fiber sensor. In most cases, the structure’s strain distribution is obtained first by the optical fiber sensor deployed on structure surface or embedded internally, and then compared with the pre-set non-destructive strain field to extract the crack information [4,5,6]. The optical fiber sensor is especially suited for large-scale crack detecting and monitoring, but its high cost and fragility have limited the application. Eddy current technique [7,8] is also developed to identify the surface or subsurface crack of the metal structures. Eddy-current inspection by Hall sensor can easily recognize the crack existence but can hardly give quantitative information. In addition, the lift-off effect is a big challenge for eddy-current detection of complicated surfaces. Since ultrasonic waves propagating in a structure would be reflected, refracted, or diffracted by defects such as cracks, the ultrasound-based inspection has become a useful technique for crack detection [9,10,11]. Ultrasonic testing is outstanding for detecting internal cracks due to its strong penetrating ability, but the drawback is the need for excitation devices and coupling agents. Acoustic emission (AE) [12,13] is another sound-based non-destructive testing method. AE refers to the phenomenon that a crack or other defect can trigger a sudden release of the stored elastic energy and thus generate a transient elastic wave. The AE signal can be collected by AE sensors (e.g., PZT patches) deployed on the structure surface, and can be analyzed to extract the crack information. Nevertheless, AE testing can hardly determine the crack shape and size, so a retesting is often needed. Infrared thermal imaging (ITM) [14,15] is an effective way of crack detection as well. Fundamentally, the thermal diffusion process would be interfered by cracks in the structure, which leads to temperature discontinuity on the observed surface. Therefore, the crack can be recognized by analyzing the recorded thermal images. The advantage of ITM is that it enables fast and full-field testing in the camera range, but the excitation devices and the infrared cameras might increase the system’s cost and complexity.

Patch antenna sensor is a novel structural health monitoring (SHM) sensor that appears in recent years. It can sense the crack in metal structures and has great application prospect due to its advantages of simple configuration, light weight, easy fabrication, low cost, etc. The idea of using patch antenna sensor to identify cracks was first proposed by Deshmukh et al. [16] in 2009. They demonstrated that the sensor’s resonant frequency dropped linearly when a crack on the ground plane propagated perpendicular to the current path. The observed sensitivity was 29.6 MHz/mm, and a sub-millimeter detection resolution could be achieved. Mohammad and Huang [17] tested the antenna sensor’s resonant frequency response to crack using a double cantilever beam and they acquired three crack sensitivities: 2.5 MHz/mm before the crack reached the patch area, 48.7 MHz/mm when the crack was underneath the patch, and 4.7 MHz/mm after the crack tip passed the patch edge. The same team also found that the patch antenna sensor can detect not only crack growth but also crack opening [18]. Later, Xu and Huang presented a four-element antenna sensor array to detect crack growth at multiple locations [19]. A wireless interrogation method was developed by implementing a light-activated RF switch, and the maximum interrogation distance of different measurement configurations could be estimated by a power budget model [20]. Yi et al. conducted an emulated crack test and a fatigue crack test to characterize the RFID-based patch antenna sensor’s performance with the presence of the crack. The experimental results show that the sensor is capable of measuring sub-millimeter crack and tracking crack propagation and that remote interrogation distance can be as far as 24 inches [21,22]. Cook et al. [23] investigated the effect of non-linear shaped (i.e., rectangular and pie-shaped) cracks on the patch antenna sensor’s resonant frequency. It is worth noting that a pie-shaped crack can decrease the resonant frequency of the radiation mode perpendicular to it but cause the other resonant frequency to increase. Cho C et al. proposed a frequency doubling scheme, which consists of a transmitting antenna, a diode-integrated matching network and a receiving antenna, for wireless interrogation of the patch antenna sensor, and measured the relationship between the antenna sensor’s resonant frequency and crack width [24]. Zhang J et al. utilized a circular patch antenna sensor with an open rectangular window for crack monitoring and presented that the antenna sensor could be useful for detecting crack depth [25].

These published studies are mainly focused on quantifying the patch antenna sensor’s behavior under the crack that coincides with the centerlines of the ground plane. No attention was paid to cracks that are more complex. Although Mohammad et al. [26] found it possible to detect crack orientation by introducing the ratio of the sensor’s two normalized resonant frequency shifts as an indicator, the quantitative relationship between the sensor’s resonant frequency and the crack information was not clarified. In this paper, we presented a more comprehensive and detailed characterization of the patch antenna sensor’s performance in sensing notch-shaped cracks. First, the crack detection mechanism was discussed, and then a patch antenna sensor was designed, simulated, fabricated and tested to understand the resonant frequency responses to various crack configurations. Besides, an algorithm was proposed for predicting both resonant frequency shifts caused by a random notch-shaped crack, which was verified by the experimental measurements. Owing to the fact that it is difficult to perform fatigue experiments to generate cracks of different characteristics for this study, we adopted a Computer Numerical Control (CNC) machine to cut slots on the sensor’s ground plane to imitate cracks in realistic metal structures. This methodology is accessible and reasonable for the lab study and can shed light upon the real scenario as well. Because wireless interrogation of patch antenna has been successfully achieved by other researchers and is not the main focus of our work, we utilized cable connection for stability and convenience in the measurement.

## 2. Principle of Operation

### 2.1. Crack Detection Mechanism of Patch Antenna Sensor

**Figure 1:**
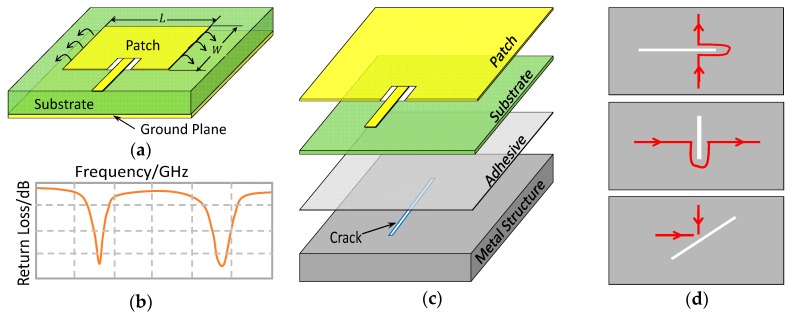
Crack detection mechanism of the patch antenna sensor. (**a**) configuration of a patch antenna; (**b**) *S*_11_ curve representing the radiation characteristics of a path antenna; (**c**) patch antenna used on metal structure as a sensor; (**d**) impact of cracks on the sensor’s current pattern.

As illustrated in Figure 1a, a rectangular patch antenna consists of a metal radiation patch, a dielectric substrate, and a conductive ground plane. These three parts form an electromagnetic (EM) resonator that works at two fundamental radiation modes: the $TM_{010}$ mode with current flowing along the patch length direction and the $TM_{001}$ mode with current flowing along the patch’s width direction. When fed with a multi-frequency EM signal, the patch antenna radiates the frequency component that matches its resonant frequency while reflects the other back. This radiation characteristic can be represented by a *S*_11_ curve shown in Figure 1b, which describes the relationship between the patch antenna’s return loss and the incident wave frequency. Accordingly, the patch antenna’s two resonant frequencies, denoted as $f_{010}$ and $f_{001}$, can be extracted from its *S*_11_ curve at the point where the return loss is a local minimum. From the transmission line theory [27], $f_{010}$ and $f_{001}$ are mainly determined by the geometric size of the radiation patch according to

(1) $$f_{mnp}=\frac{c}{2\pi \sqrt{\varepsilon_{re}}}\sqrt{{\left(\frac{m\pi }{h}\right)}^{2}+{\left(\frac{n\pi }{L}\right)}^{2}+{\left(\frac{p\pi }{W}\right)}^{2}},\;\left(m=0;n=0,1;p=0,1\right)$$

where *c* is the velocity of light in vacuum, $\varepsilon_{re}$ is the effective dielectric constant of the substrate, h is the thickness of the substrate, and L and W are the geometric length and width of the patch, respectively.

Considering the patch antenna’s configuration and the fact that any good conductor can be a ground plane, we can create a SHM sensor by bonding a substrate and a radiation patch to the surface of metal structure using adhesive, as shown in Figure 1c. In this case, the metal structure serves as the ground plane, and the formed patch antenna sensor can perceive certain physical quantity changes of the metal structure. For example, a surface crack in the metal structure (see Figure 1c) would cause a local conductivity loss and therefore disturb the current path on the ground plane, leading to a resonant frequency shift of the patch antenna sensor. As demonstrated in Figure 1d, a crack parallel to the patch length would significantly increase the current path of $TM_{010}$ mode and thus change $f_{010}$, and a crack parallel to the patch width would do the same to $TM_{001}$ mode and $f_{001}$. A crack with a certain inclination would affect both radiation modes and resonant frequencies. Characterizing the patch antenna sensor’s resonant frequency response to crack would give us means to inversely identify the crack information from the measured resonant frequencies.

### 2.2. Analytical Expression of Antenna Sensor’s Current Distribution

**Figure 2:**
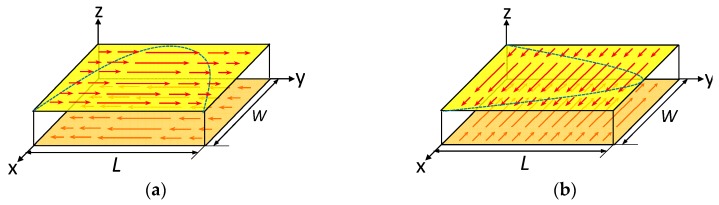
Current distribution of different antenna radiation modes. (**a**) current density of the $TM_{010}$ mode; (**b**) current density of the $TM_{001}$ mode.

To understand the antenna sensor’s behavior with a cracked ground plane, the current density on its ground plane and the patch was derived first. For the coordinate system shown in Figure 2, the EM field inside the patch antenna could be expressed as Equation (2) according to the ‘cavity model’ and the vector potential method [27,28]:

$$E_{z}=-j\frac{\left(k^{2}-k_{z}{}^{2}\right)}{\omega \mu \epsilon }A_{mnp}\cos\left(k_{x}x\right)\cos\left(k_{y}y\right)\cos\left(k_{z}z\right)$$

$$E_{x}=-j\frac{k_{x}k_{z}}{\omega \mu \epsilon }A_{mnp}\sin\left(k_{x}x\right)\cos\left(k_{y}y\right)\sin\left(k_{z}z\right)$$

(2) $$E_{y}=-j\frac{k_{y}k_{z}}{\omega \mu \epsilon }A_{mnp}\cos\left(k_{x}x\right)\sin\left(k_{y}y\right)\sin\left(k_{z}z\right)$$

$$H_{z}=0$$

$$H_{x}=\frac{k_{y}}{\mu }A_{mnp}\cos\left(k_{x}x\right)\sin\left(k_{y}y\right)\cos\left(k_{z}z\right)$$

$$H_{y}=-\frac{k_{x}}{\mu }A_{mnp}\sin\left(k_{x}x\right)\cos\left(k_{y}y\right)\cos\left(k_{z}z\right)$$

where ω is the angular frequency of time-harmonic field, while μ and ϵ are the permeability and the dielectric constant of the substrate, respectively. $A_{mnp}$ is the amplitude of the introduced vector potential for $TM_{mnp}$ mode. $k_{x}$, $k_{y}$, $k_{z}$ are the wavenumbers along the *x*, *y*, *z* directions, and are calculated as:

$$k_{z}=\frac{m\pi }{h},m=0,1,2,\ldots$$

(3) $$k_{y}=\frac{n\pi }{L},n=0,1,2,\ldots$$

$$k_{x}=\frac{p\pi }{W},p=0,1,2,\ldots$$

For the $TM_{010}$ mode, $k_{x}=k_{z}=0$ and $k_{y}=\pi /L$, so its EM field components should be written as (where $E_{0}$ and $H_{0}$ are the maximum values of sinusoidal E and H)

$$E_{z}=E_{0}\cos\left(\frac{\pi }{L}y\right)$$

(4) $$H_{x}=H_{0}\sin\left(\frac{\pi }{L}y\right)$$

$$E_{x}=E_{y}=H_{z}=H_{y}=0$$

For the $TM_{001}$ mode, $k_{y}=k_{z}=0$ and $k_{x}=\pi /W$, and its EM field components should be written as:

$$E_{z}=E_{0}\cos\left(\frac{\pi }{W}x\right)$$

(5) $$H_{y}=H_{0}\sin\left(\frac{\pi }{W}x\right)$$

$$E_{x}=E_{y}=H_{z}=H_{x}=0$$

After the EM field is determined, the current density on the ground plane (or the patch) can be calculated based on [29]:

(6) $$J=\vec{n}\times H$$

where $\vec{n}$ is the unit normal vector of the ground plane (or the patch). Since H for $TM_{010}$ mode is along the x direction, the corresponding current density is along the y direction and should be expressed as:

(7) $$J_{y}=J_{010}\sin\left(\frac{\pi }{L}y\right)$$

Similarly, the current density for $TM_{001}$ mode is along the x direction and the analytical expression is

(8) $$J_{x}=J_{001}\sin\left(\frac{\pi }{W}x\right)$$

The current flows for $TM_{010}$ mode and $TM_{001}$ mode are depicted in Figure 2a,b where the length of the arrows indicates the magnitude of the current density. It is obvious that the maximum current appears in the middle of the ground plane and decreases gradually to zero at the edges.

## 3. Sensor Design and Simulation

### 3.1. Sensor Design Parameters

**Figure 3:**
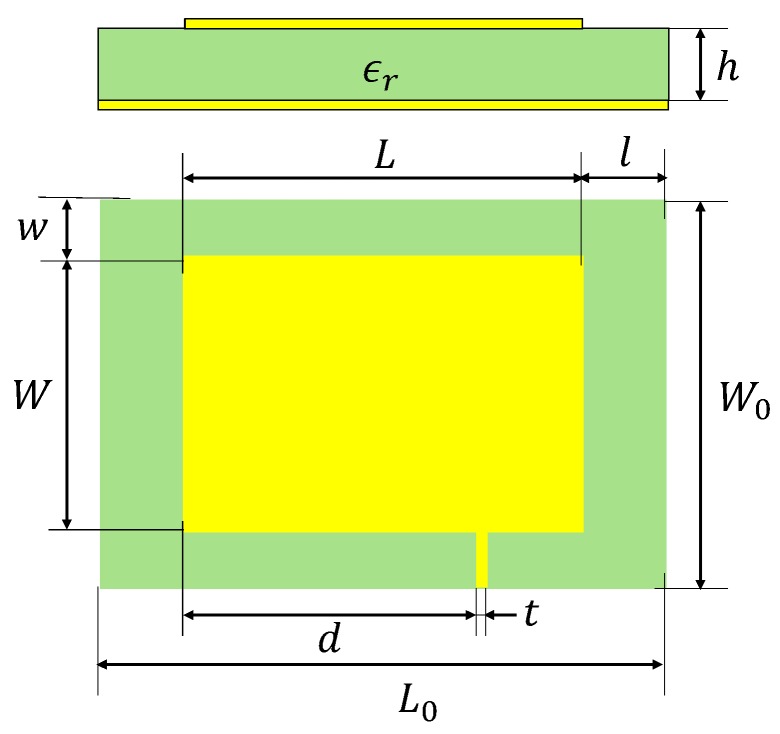
Antenna sensor design parameters.

As shown in Figure 3, a rectangular patch antenna sensor was designed using the procedure described in [16]. The FR4 plane with a thickness of 0.5 mm and a dielectric constant of 4.4 was chosen as the substrate. The initial resonant frequencies were selected to be $f_{010}$ = 1.8 GHz and $f_{001}$ = 2.5 GHz because the frequency range of our vector network analyzer (VNA) is 300 kHz–3 GHz. For the selected substrate, this resulted in a 39.62 mm long and 27.89 mm wide radiation patch, which were rounded up to be 40 mm and 28 mm for the convenience of manufacturing. In addition, the patch is fed at a proper position with a transmission line, through which the incident signal can be applied to excite the radiation modes. The detailed parameters for each part of the designed antenna sensor can be found in Table 1.

### 3.2. Simulation Model

**Figure 4:**
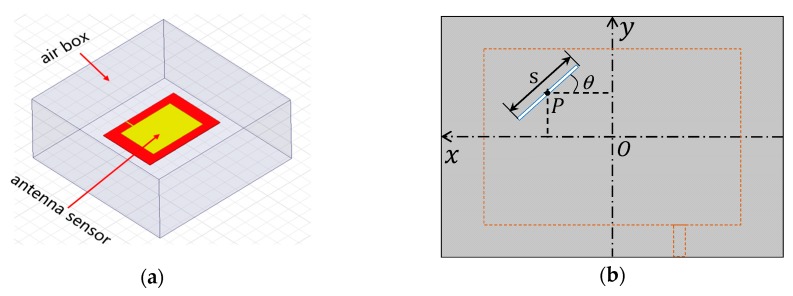

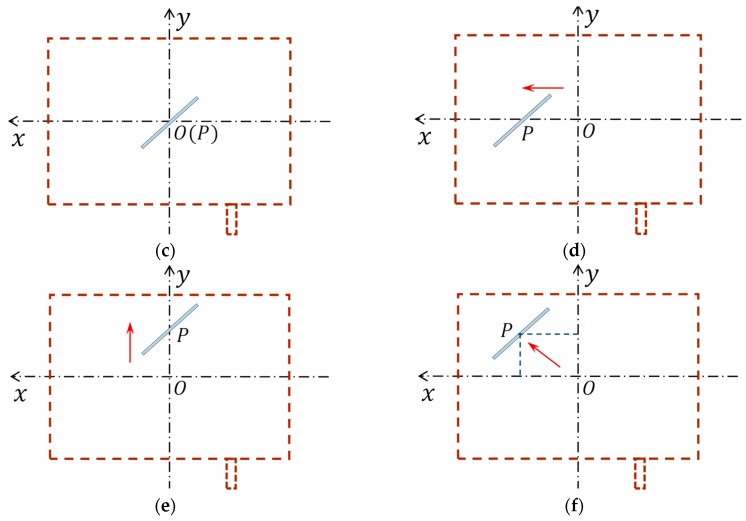
Simulation model. (**a**) entire model in HFSS^TM^; (**b**) quantitative description of the crack; (**c**) cracks located at the center of the ground plane; (**d**) crack position moving leftwards; (**e**) crack position moving upwards; (**f**) crack position moving leftwards and upwards simultaneously.

The designed sensor was modeled in commercial EM simulation software HFSS^TM^ (Ansoft, Pittsburgh, PA, USA) to characterize its crack sensing ability. As shown in Figure 4a, the entire antenna sensor is confined in an air box whose surfaces are set as radiation boundaries. The patch and the ground plane are treated as perfect electrical conductors. The antenna sensor is excited at the end of the transmission line with a 50 Ω lumped port. On the ground plane, a 0.6 mm wide slot was introduced to imitate the crack. Since the crack could appear randomly, three parameters were predefined to quantitatively describe it. In the coordinate system shown in Figure 4b, a crack is represented by a line-shaped slot. The midpoint P and the length s of the slot denote the crack position and crack length, respectively, and the crack orientation is defined as the angle θ between the slot and the *x*-axis. As such, an arbitrary crack on the ground plane can be presented by three parameters—the position P, length s, and orientation θ.

In order to study the resonant frequency response to crack, the antenna sensor was modeled with the following procedure. Firstly, the crack position P was fixed at the center of the ground plane to investigate the variation of resonant frequency with the crack length and orientation change (see Figure 4c)). After that, the same investigation was conducted by moving the crack position P leftwards along the *x*-axis (see Figure 4d) and upwards along the *y*-axis (see Figure 4e), respectively, with a step of 3 mm. Finally, the crack position P was moved simultaneously in both directions (see Figure 4f), and the corresponding resonant frequency shifts were analyzed. In the entire modeling process, the crack length increased with a step of 1 mm and the crack profile was limited inside the area under the patch. Considering the symmetry of the antenna geometry and the current density, the crack orientation θ was assigned to vary from 0° to 90° with an increment of 15°.

### 3.3. Simulated Results

**Figure 5:**
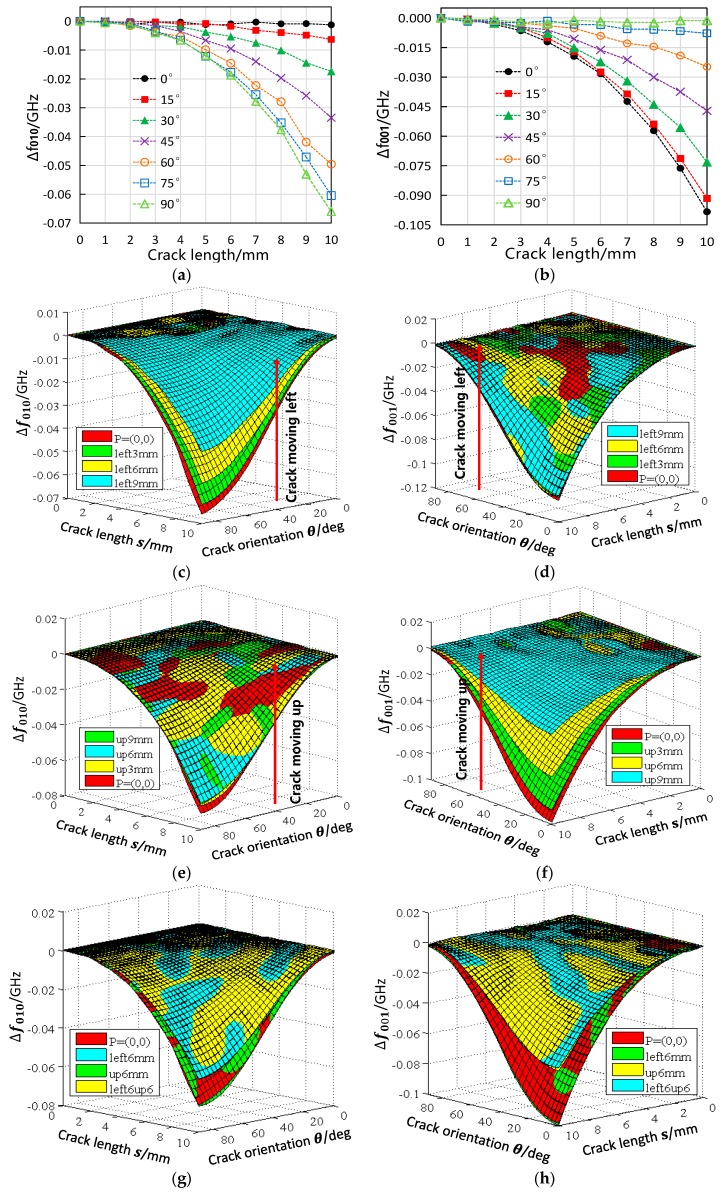
Simulated results. (**a**) $f_{010}$ and (**b**) $f_{001}$ response to cracks at the center of the ground plane; (**c**) $f_{010}$ and (**d**) $f_{001}$ response with the crack position moving leftwards; (**e**) $f_{010}$ and (**f**) $f_{001}$ response with the crack position moving upwards; (**g**) $f_{010}$ and (**h**) $f_{001}$ response with the crack position moving leftwards and upwards simultaneously.

**Figure 6:**
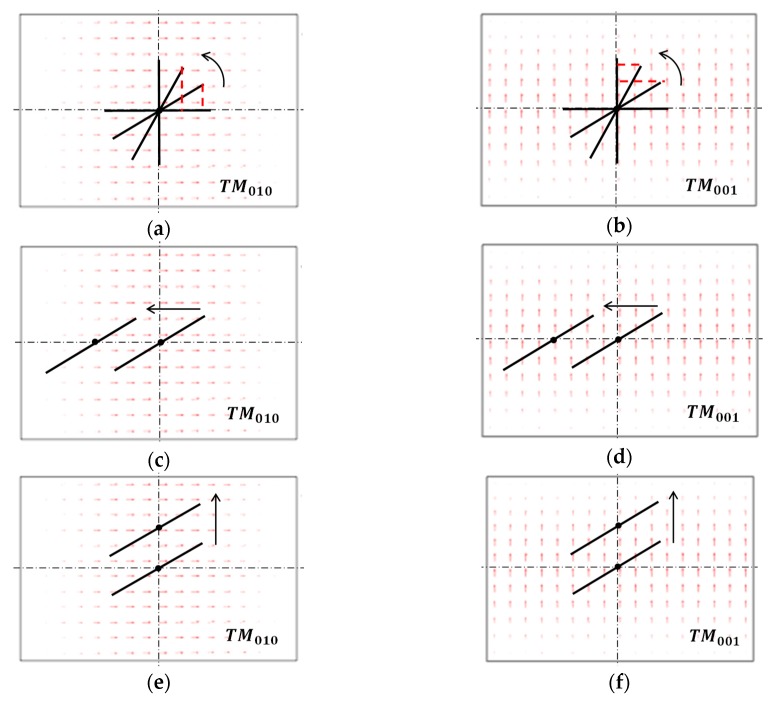
Interaction between the crack and the sensor’s current flow. (**a**,**b**) cutting effect of the cracks at ground plane center on both current flows; (**c**,**d**) cutting effect of the crack moving leftwards; (**e**,**f**) cutting effect of the crack moving upwards.

The simulated response of the sensor’s resonant frequency to the cracks at the coordinate origin is shown in Figure 5a,b. When the crack orientation θ is 0°, $f_{010}$ almost remains constant. In other directions, $f_{010}$ drops with the increase of the crack length, and the closer θ approaches 90° the greater the drop is. Conversely, $f_{001}$ is unchanged when the crack orientation is 90° but declines fastest with the crack growth in 0° direction. These observations could be explained by the interaction between the crack and the current flow on the ground plane. As shown in Figure 6a,b, the cracks in 0° direction or 90° direction only perturbs the current of one of the two modes while has no influence on the other, therefore causing only one of the resonant frequencies to decrease. When the crack of the same length gradually varies from 0° direction to 90° direction, its effective cutting length on $TM_{010}$ mode current gets bigger (see Figure 6a) whereas that on $TM_{001}$ mode drops down (see Figure 6b). This accounts for the different rate in resonant frequency reduction resulted from the crack orientation change.

For the crack configuration in Figure 4d, the simulated results are plotted in Figure 5c,d. The surface ‘P=(0,0)’ represents the sensor’s resonant frequency response to the crack whose position is at the coordinate origin, and the other surfaces are the corresponding results of the cracks moving leftwards by 3, 6, and 9 mm. In the process of the crack moving leftwards, the trend of the $f_{010}$ change keeps constant but the amplitude of the change decreases in sequence; the $f_{001}$ response remains approximately the same as when the crack is at the coordinate origin. The reason for this is shown in Figure 6c,d. When moving to the left, the crack enters the low-density area of $TM_{010}$ mode current from the high-density area. As a result, the cutting effect of the crack on $TM_{010}$ mode current is gradually weakened and the amount of $f_{010}$ shift declines accordingly. In comparison, the magnitude of $f_{001}$ shift does not change because the crack is still in the high-density area of $TM_{001}$ mode current when moving along the *x*-axis.

When the crack moves upwards as in Figure 4e, the antenna sensor’s resonant frequency responses are shown in Figure 5e,f. In this case, the response of $f_{010}$ to the crack stays unchanged all the way while the shift of $f_{001}$ gets weaker. Similar to the scenario where the crack moves leftwards, this is caused by the current density change of the region being cut by the crack (see Figure 6e,f).

To understand the sensor’s resonant frequency behavior with the crack position changes shown in Figure 4f, the results of four selected crack configurations, i.e., crack at the coordinate origin, crack moving to the left by 6 mm, crack moving up by 6 mm, and crack moving leftwards and upwards simultaneously by 6 mm, are compared in Figure 5g,h. It can be seen that the $f_{010}$ response of ‘left6up6’ is almost the same as that of ‘left6mm’ and the $f_{001}$ response of ‘left6up6’ and ‘up6mm’ roughly overlap. This indicates that crack movements along the *x*-axis and *y*-axis independently affect the resonant frequency $f_{010}$ and $f_{001}$, respectively.

## 4. Algorithm for Predicting the Crack-Caused Resonant Frequency Shifts

### 4.1. Definition of the Current Cutting Effect Coefficient φ

According to the above simulation and analysis, the influence of the crack on the antenna sensor’s resonant frequencies is governed by the crack’s cutting effect on the current flow of both modes. Generally, this cutting effect leads to the resonant frequency decrease, and the decrease becomes more significant when the cutting effect is more intense. The intensity of this cutting effect is related to the crack position, length, and orientation, so we introduced a coefficient φ  to comprehensively describe it. The definition of φ is

**Figure 7:**
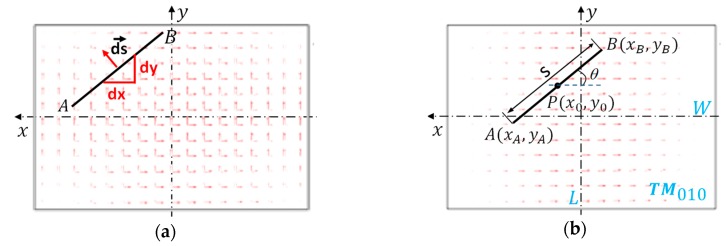
Definition of crack’s cutting effect intensity φ on the antenna current. (**a**) definition; (**b**) calculation of ${\varphi \;}_{010}$.

As shown in Figure 7a, line AB represents an arbitrary crack, and $d\vec{s}$ is one element on it. The element’s cutting effect intensity dφ can be expressed by vector point multiplication as:

(9) $$d\varphi =d\vec{s}\cdot \vec{j_{s}}$$

where $\vec{j_{s}}$ is the current density vector at the element point. Therefore, the crack’s total cutting effect intensity φ  is the integration of dφ  along the entire crack path, i.e.,

(10) $$\varphi =\int_{A}^{B}\vec{j_{s}}\cdot d\vec{s}$$

The calculation of formula (10) is illustrated in Figure 7b. Denote the crack position, length and orientation as $P\left(x_{0},y_{0}\right)$, s and θ, respectively, and the coordinates of A and B can be written as

(11) $$A\left(x_{A},y_{A}\right)=\;A\left(x_{0}-\frac{s\cos\theta }{2},y_{0}-\frac{s\sin\theta }{2}\right)$$

(12) $$B\left(x_{B},y_{B}\right)=\;B\left(x_{0}+\frac{s\cos\theta }{2},y_{0}+\frac{s\sin\theta }{2}\right)$$

From Equations (7) and (8), the current distribution of two modes in the coordinate system of Figure 7b should be expressed as

(13) $${\vec{J}}_{010}\left(x,y\right)=J_{010}\sin\left[\frac{\pi }{L}\left(x+\frac{L}{2}\right)\right]{\vec{e}}_{x}$$

(14) $${\vec{J}}_{001}\left(x,y\right)=J_{001}\sin\left[\frac{\pi }{W}\left(y+\frac{W}{2}\right)\right]{\vec{e}}_{y}$$

where $J_{010}$ and $J_{001}$ are the maximum values of the current densities of $TM_{010}$ and $TM_{001}$ mode. ${\vec{e}}_{x}$ and ${\vec{e}}_{y}$ are the unit vectors along the *x*-axis and *y*-axis. The cutting effect intensity of crack AB on $TM_{010}$ mode current is then

(15) $${\varphi \;}_{010}=\int_{A}^{B}{\vec{J}}_{010}\left(x,y\right)\cdot \vec{ds}=\int_{y_{A}}^{y_{B}}J_{10}\sin\left[\frac{\pi }{L}\left(x+\frac{L}{2}\right)\right]dy$$

For 0° ≤θ< 90°, the integration path AB can be written as

(16) $$y=\tan\theta x+y_{0}-x_{0}\tan\theta$$

By changing the integral variable to dx, Equation (15) can be calculated as

(17) $${\varphi \;}_{010}=\int_{x_{A}}^{x_{B}}J_{010}\sin\left[\frac{\pi }{L}\left(x+\frac{L}{2}\right)\right]\tan\theta dx=\frac{J_{10}L\tan\theta }{\pi }\left(\sin\frac{\pi x_{B}}{L}-\sin\frac{\pi x_{A}}{L}\right)$$

For θ= 90°, the crack is perpendicular to the current of $TM_{010}$ mode. Therefore, its cutting effect intensity should be ${\varphi \;}_{010}=sJ_{010}$. Overall, the cutting effect coefficient of crack AB on $TM_{010}$ mode current is defined as

(18) $$\varphi {\;}_{010}=sJ_{010}\;\left(\theta =90^{\circ }\right)$$

$$\varphi {\;}_{010}=\frac{J_{010}L\tan\theta }{\pi }\left(\sin\frac{\pi x_{B}}{L}-\sin\frac{\pi x_{A}}{L}\right),\;(0^{\circ }\leq \theta <90^{\circ })$$

Following a similar process, the cutting effect coefficient of crack AB on $TM_{001}$ mode current can be derived as

(19) $${\varphi \;}_{001}=sJ_{001}\;\left(\theta =0^{\circ }\right)$$

$${\varphi \;}_{001}=\frac{J_{001}W}{\pi \tan\theta }\left(\sin\frac{\pi y_{B}}{W}-\sin\frac{\pi y_{A}}{W}\right),\;(0^{\circ }<\theta \leq 90^{\circ })$$

### 4.2. Relationship between Resonant Frequency Shift and Coefficient φ

**Figure 8:**
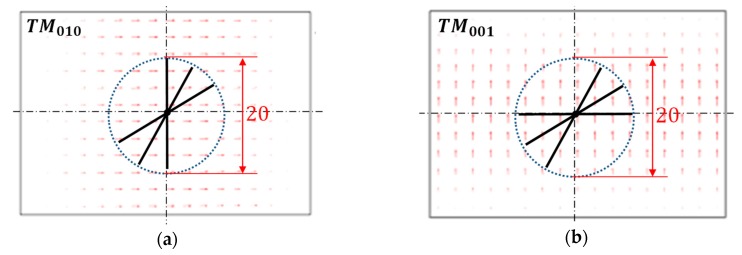

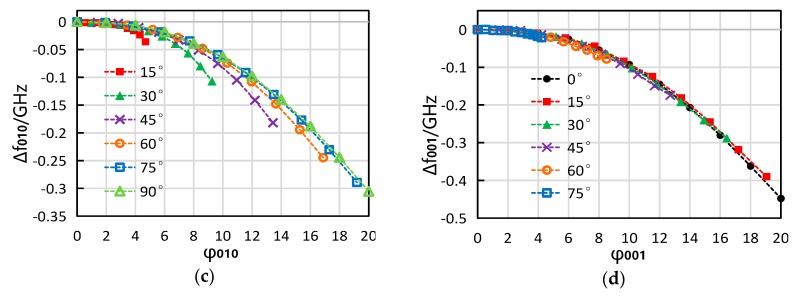
Investigation on the relationship between the sensor’s resonant frequency shift and coefficient φ . (**a**,**b**) simulation model; (**c**) $\Delta f_{010}–\varphi_{010}$ relationship; and (**d**) $\Delta f_{001}–\varphi_{001}$ relationship.

After coefficient φ was defined, we investigated the relationship between the sensor’s resonant frequency shift and φ using the simulation model shown in Figure 8a,b. The cracks were located at the coordinate origin, growing from 0 mm to 20 mm in six orientations. The obtained Δf–φ relationship are presented in Figure 8c,d, in which the vertical axis is the Δf value from HFSS and the horizontal axis is the φ value calculated by Mathematica. It can be seen that $\Delta f_{001}–\varphi_{001}$ relationship keeps the same regardless of the crack configuration. By contrast, $\Delta f_{010}–\varphi_{010}$ relationship of each orientation remains unified when $\varphi_{010}$ is small but begins to deviate when φ gets to a certain value. Such a difference between $\Delta f_{010}–\varphi_{010}$ and $\Delta f_{001}–\varphi_{001}$ is probably related to the antenna sensor’s feeding way and feeding position.

### 4.3. Process of the Algorithm

**Figure 9:**
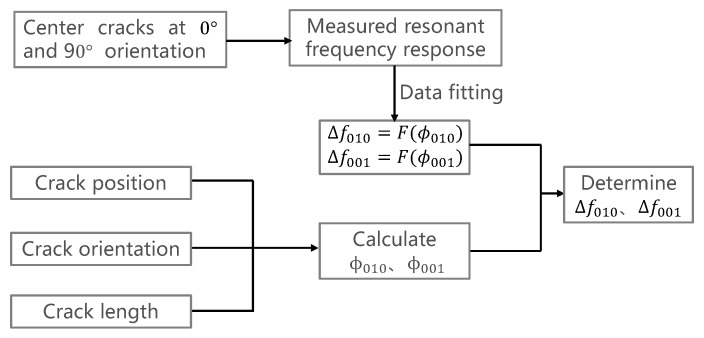
Algorithm for predicting the crack-caused resonant frequency shifts.

Based on the above analysis, we present an algorithm for predicting resonant frequency shifts caused by an arbitrary crack. The fundamental idea is to first calculate the φ of the crack and then to get the resonant frequency shifts according to the Δf–φ relationship. In this paper, we select the $\Delta f_{010}–\varphi_{010}$ relationship of the crack at 90° orientation and the $\Delta f_{001}–\varphi_{001}$ relationship of the crack at 0° orientation as standard Δf–φ relationships of two radiation modes. The process of the algorithm is shown in Figure 9, which will be validated by experimental data in the following content.

## 5. Experimental Validation

### 5.1. Sensor Fabrication and Experiment Setup

**Figure 10:**
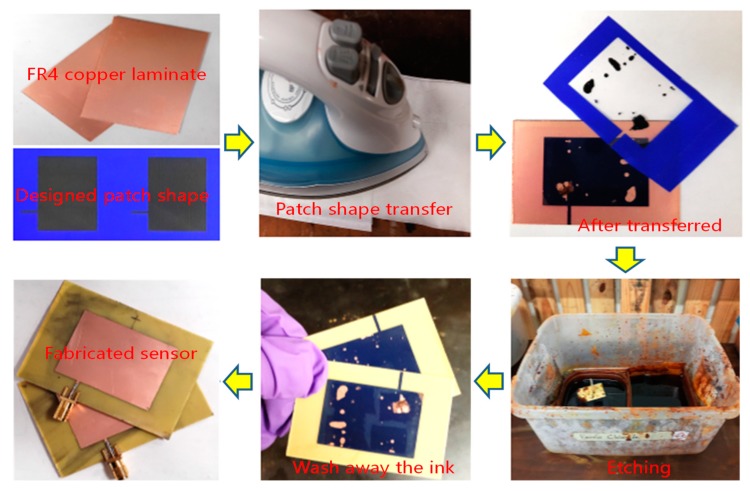
Fabricating process of the antenna sensor.

The antenna sensor was fabricated by the chemical etching process shown in Figure 10. First, a 0.6 mm thick FR4 copper clad laminate was cut into 64mm×44mm pieces. The designed patch shape was then printed on a PCB pattern transfer paper and transferred to one side of the laminate, followed by dipping it into a ferric chloride solution to etch the unwanted copper. After the etching is done, acetone was used to wash off the ink covering the patch and the SMA connector was soldered.

**Figure 11:**
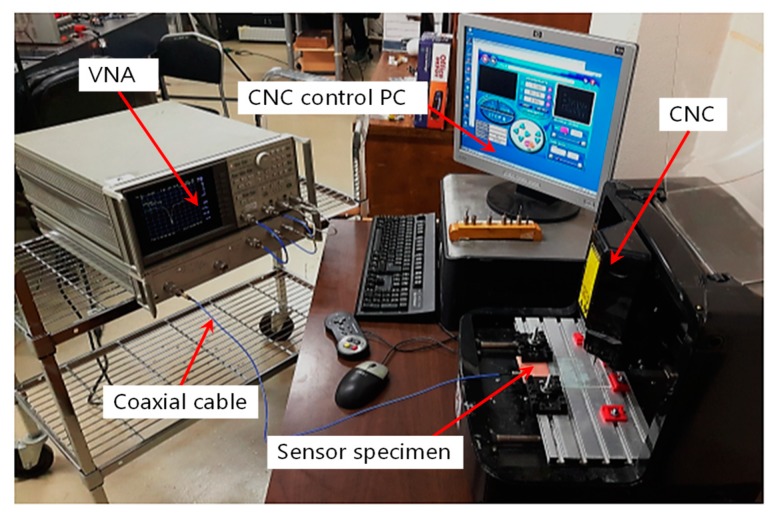
Experiment setup.

An experimental setup (see Figure 11) was established to test the antenna sensor’s crack sensing behavior. The sensor specimen was clamped on the work plane of a CNC machine (Click N Carve 84015, Rockler, Medina, MN, USA) and connected to a vector network analyzer (VNA) (Hewlett Packard 85047A, Keysight, Santa Rosa, CA, USA) through the SMA connector and the coaxial cable. The CNC machine was used to cut the cracks of different lengths and orientations at different positions of the sensor’s ground plane. For each tested crack configuration, the step of the crack length increase was set to be 2 mm. This was achieved by controlling the blade to cut into the ground plane at the midpoint of the scheduled crack profile (i.e., crack position P) and then move 1 mm further to the left and the right, respectively. After the cutting was done, the sensor specimen was removed from the CNC machine and the actual crack length was measured using a ruler before the resonant frequencies were acquired by the VNA. The VNA was set to sweep over a span of 40 MHz with 1601 frequency points in every measurement, which results in a 25 kHz frequency resolution.

### 5.2. Experiment Results

**Figure 12:**
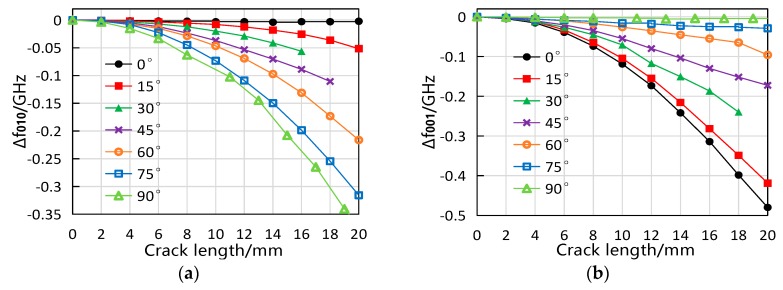
Measured resonant frequency response to cracks located at the center of the ground plane. (**a**) $f_{010}$ results and (**b**) $f_{001}$ results.

The measured data of sensors with cracks located at the center of the ground plane is plotted in Figure 12. Cracks in seven directions were tested, and the length for all the cracks was increasing from 0 to 20 mm with an interval of 2 mm. As predicted by the simulation, both resonant frequencies decreased with the crack growth, and the equal-length cracks in different orientations led to different amount of frequency shifts. It could be noticed that some data points were missing because the *S*_11_ curve degraded (which only showed one or no resonant frequency peak) at certain crack lengths and directions. This was probably due to the impedance mismatch of the antenna sensor in such crack circumstances.

**Figure 13:**
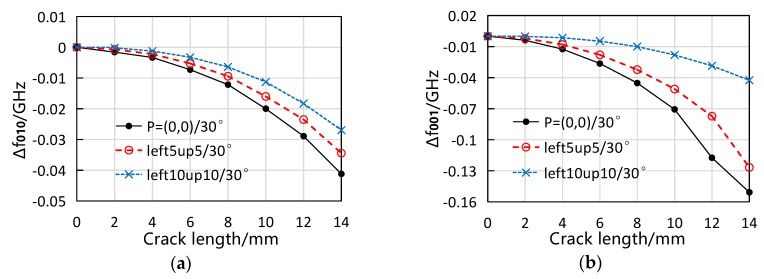

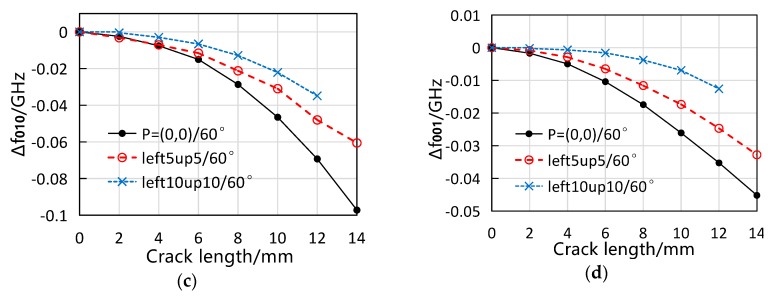
Measured resonant frequency response with the crack moving to P=(5,5) and P=(10,10). (**a**) $f_{010}$ and (**b**) $f_{001}$ response at 30° direction; (**c**) $f_{010}$ and (**d**) $f_{001}$ response at 60° direction.

To efficiently validate the effect of crack movement on the antenna sensor’s resonant frequencies, the crack position was moved away from the coordinate origin to P=(5,5) and P=(10,10) respectively, and only two typical directions, i.e., 30° and 60°, were tested. For the convenience of comparing the results of different cracks, the crack length was increased from 0 mm to 14 mm with an increment of 2 mm. The observed resonant frequency responses are shown in Figure 13. Some data points were missing because the crack tips were out of the patch area. It is obvious that, for either $f_{010}$ or $f_{001}$, the rate of the resonant frequency decrease becomes smaller as the crack position moves further away from the coordinate origin. Qualitatively, this behavior agrees with the effect of crack position movements on the resonant frequency response revealed by simulation. However, such phenomenon is not prominent when the crack is short (e.g., less than 4 mm in length). This might be contributed by the fact that the resonant frequency shifts are so small at the beginning of the crack growth that the measurement errors (either in measuring the crack length or the resonant frequencies) could have significant influence.

### 5.3. Case Study—Validation of the Proposed Algorithm

In order to evaluate the effectiveness of the proposed algorithm, the fundamental idea is to measure the resonant frequency shifts of the antenna sensor with an arbitrary crack, and then compare the results with the corresponding values predicted by the algorithm. Since the crack could not be really arbitrary in the test, we selected two crack configurations to conduct the case study. One crack was located at P=(7,3), growing in the direction of θ= 40°; the other was located at P=(4,2), growing in the direction of θ= 55°. The crack length was assigned to increase 2 mm per step to collect more data points.

Prior to the measurement, the $\Delta f_{010}=F\left(\varphi_{010}\right)$ was obtained by cubic polynomial fitting the measured data of the crack that was located at the coordinate origin and propagates in 90° direction (i.e., the ‘90°’ curve in Figure 12a) Similarly, the $\Delta f_{001}=F\left(\varphi_{001}\right)$ was the cubic polynomial fitting of the measured data of the crack located at the coordinate origin and propagating in 0° direction (i.e., the ‘0°’ curve in Figure 12b. The acquired functions are shown as the Equations (20) and (21), and the corresponding $R^{2}$ values are 0.9999 and 0.9998, respectively. To calculate the predicted resonant frequency shifts, the coefficient $\varphi_{010}$ and $\varphi_{001}$ of a certain crack were calculated first according to the Equations (18) and (19), and then $\Delta f_{010}$ and $\Delta f_{001}$ could be gained from the Equations (20) and (21).

(20) $$\Delta f_{010}=F\left(\varphi_{010}\right)=4.98\times 10^{-6}\varphi_{010}{}^{3}-0.001\varphi_{010}{}^{2}+0.00015\varphi_{010}-0.0008$$

(21) $$\Delta f_{001}=F\left(\varphi_{001}\right)=1.44\times 10^{-5}\varphi_{001}{}^{3}-0.0016\varphi_{001}{}^{2}+0.0033\varphi_{001}-0.0021$$

**Figure 14:**
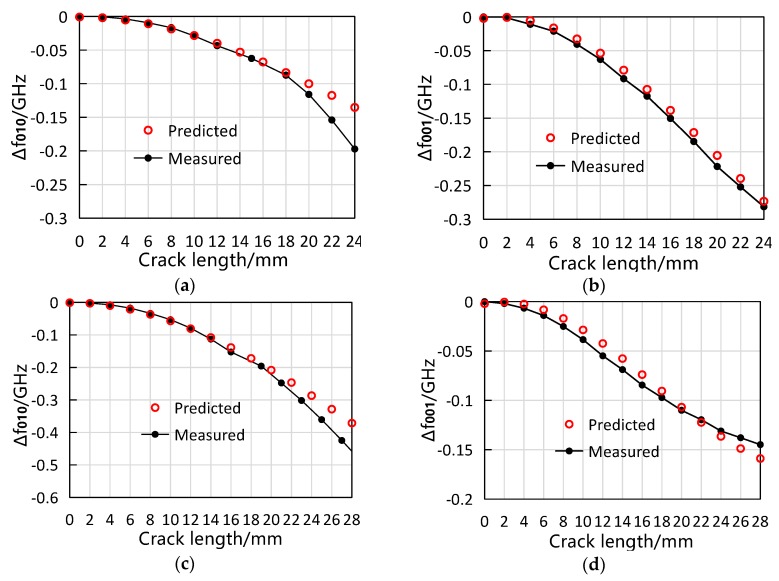
Evaluation of the proposed algorithm. (**a**,**b**) comparison between the measured and the predicted resonant frequency response for case P=(7,3) and θ= 40°; (**c**,**d**) comparison between the measured and the predicted resonant frequency response for case P=(4,2) and θ= 55°.

The comparison between the measured resonant frequency shifts and their predicted counterparts are shown in Figure 14. At most data points that are observed, the prediction is in good agreement with the measurement although a slight difference can be seen. The discrepancy might come from the errors in the process of measuring the crack length and extracting the resonant frequency from the *S*_11_ curve. Another source of the discrepancy might be the ideal assumption in the proposed algorithm. When calculating the predicted resonant frequency, the coefficient φ  is based on the current density function (13) and (14), which should be slightly different from the real current density of the tested antenna sensor because of the antenna’s fringing effect in practical scenario. It is also observed that, for $f_{010}$ in both case studies, the discrepancy between the predicted resonant frequency shift and the measured one becomes considerable when the crack is longer than 20 mm. This can be explained by the inconsistency of $\Delta f_{010}$–$\varphi_{010}$ relationships (as shown in Figure 8c): since we take the $\Delta f_{010}$–$\varphi_{010}\varphi_{010}$ relationship of the crack at 90° direction as the standard in the algorithm, the predicted $\Delta f_{010}$ for the crack at other directions would not be accurate if the crack’s $\varphi_{010}$ is relatively large. Moreover, it can be anticipated that the algorithm-predicted $\Delta f_{010}$ would be more precise for the crack whose orientation is closer to 90°. Generally, the proposed algorithm works well in predicting the resonant frequency shifts but may lose the accuracy in predicting $\Delta f_{010}$ of long cracks. This performance is likely to be improved by modifying the $\Delta f_{010}$–$\varphi_{010}\varphi_{010}$ in the future study.

## 6. Conclusions

This study characterized the response of a patch antenna sensor’s resonant frequency to notch-shaped cracks. Both the simulation and experiment demonstrate that the crack position, length and orientation exert influence on the resonant frequencies in a way that could be represented by the interaction between the crack and the current distribution of the sensor’s ground plane. As a result, we presented an algorithm for predicting the resonant frequency shifts caused by a random notch-shaped crack. The experimental tests show that this algorithm works well in most cases but might be inaccurate in predicting $\Delta f_{010}$ when the crack is of a considerable length. Based on this study, the behavior of patch antenna sensor’s resonant frequencies in identifying notch-shaped cracks could be substantially understood and quantitatively described, which contributes to the research and development of patch antenna sensors for SHM purposes. The subsequent work in this field should be focused on developing the inversion algorithm for crack identification and monitoring.

## Figures and Tables

**Table 1:** Detailed parameters of the designed antenna sensor.

Symbol	Physical Quantity	Selected Value
$f_{010}$, $f_{001}$	Designed initial resonant frequencies	1.80 GHz, 2.54 GHz
$\epsilon_{r}$	Substrate dielectric constant	4.4
h	Substrate thickness	0.5 mm
L	Radiation patch length	40 mm
W	Radiation patch width	28 mm
t	Transmission line width	1 mm
d	Transmission line position	29.5 mm
$L_{0}$	Length of substrate/ground plane	64 mm
$W_{0}$	Width of substrate/ground plane	44 mm
l	Horizontal distance from patch edge to substrate edge	12 mm
w	Vertical distance from patch edge to substrate edge	8 mm
